# Corps étranger au niveau de la trompe

**DOI:** 10.11604/pamj.2015.22.237.7963

**Published:** 2015-11-13

**Authors:** Manel Yahia, Issam M'sakni

**Affiliations:** 1 Service d'Anatomie et de Cytologie Pathologiques, Hôpital Militaire de Tunis, Tunisie

**Keywords:** Trompe, corps étranger, fallopian tube, foreign body

## Image en medicine

Pièce de salpingectomie réalisée par laparotomie exploratrice pour pélvi-péritonite d'une femme de 27 ans, sans antécédents pathologiques et suivie pour stérilité primaire. En per opératoire, la trompe était dilatée et recouverte de fausses membranes. A l'ouverture de la pièce, la trompe comportait une bougie déformée de 17 cm de long. La reprise de l'interrogatoire a révélé que la patiente a consulté pour sa stérilité un charlatan qui lui a conseillé de mettre une bougie dans ses voies génitales.

**Figure 1:**
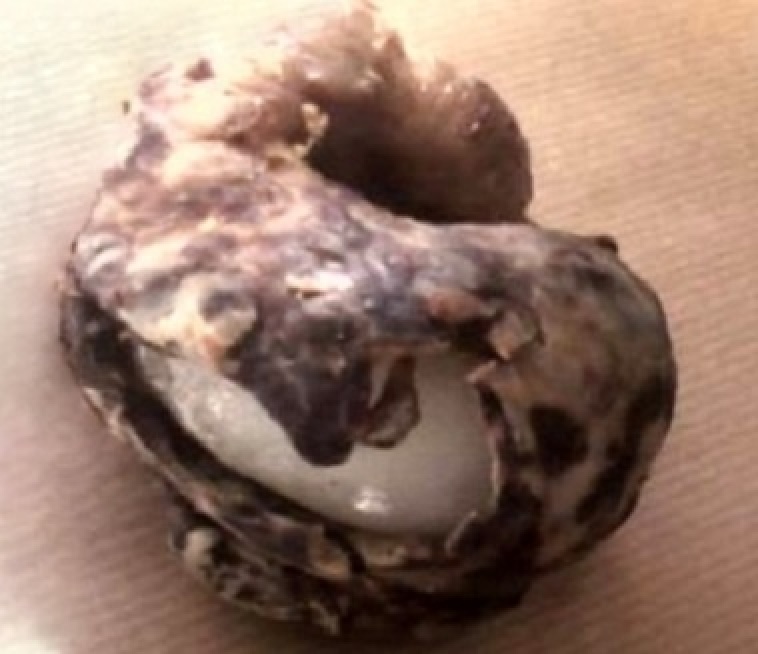
Pièce de salpingectomie montrant une trompe boudinée recouverte de fausses membranes; à l'ouverture de la pièce, on note la présence d'un corps étranger blanchâtre correspondant à une bougie

